# Sex differences in bladder cancer: understanding biological and clinical implications

**DOI:** 10.1186/s13293-025-00715-6

**Published:** 2025-05-13

**Authors:** Prakash Chaudhary, Biplab Singha, Hany A. Abdel-Hafiz, Maria Velegraki, Debasish Sundi, Swati Satturwar, Anil V. Parwani, Sergei I. Grivennikov, Sungyong You, Helen S. Goodridge, Qin Ma, Yuzhou Chang, Anjun Ma, Bin Zheng, Dan Theodorescu, Zihai Li, Xue Li

**Affiliations:** 1 Department of Medicine and Department of Biomedical Sciences, Cedars-Sinai Medical Center, Los Angeles, CA USA; 2 Samuel Oschin Comprehensive Cancer Institute, Cedars-Sinai Medical Center, Los Angeles, CA USA; 3 Department of Urology, Cedars-Sinai Medical Center, Los Angeles, CA USA; 4 Pelotonia Institute for Immuno‑Oncology, The Ohio State University Comprehensive Cancer Center, Columbus, OH USA; 5 Department of Urology, Division of Urologic Oncology, The Ohio State University, Comprehensive Cancer Center Board of Governors, Columbus, OH USA; 6 Department of Pathology, Wexner Medical Center at The Ohio State University, Columbus, OH 43210 USA; 7 Board of Governors Regenerative Medicine Institute, Cedars-Sinai Medical Center, Los Angeles, CA 90048 USA; 8 Department of Biomedical Informatics, College of Medicine, The Ohio State University, Columbus, OH USA

**Keywords:** Sex chromosomes, Sex hormones, Non-canonical sex-biasing factors, Urothelial carcinoma, Precision medicine

## Abstract

Bladder cancer (BC) remains a significant global health concern, with substantial sex and racial disparities in incidence, progression, and outcomes. BC is the sixth most common cancer among males and the seventeenth most common among females worldwide. Over 90% of BC cases are urothelial carcinoma (UC) with high degrees of pathological heterogeneity. Molecular subtyping of BC has also revealed distinct luminal, basal, and neuroendocrine subtypes, each with unique genetic and immune signatures. Emerging research uncovers the biasing effects of the sex hormones with androgens increasing BC risk through both tumor cell intrinsic and extrinsic mechanisms. The sex chromosomes, including both the X and Y chromosomes, also contribute to the sex differences in BC. The effect of sex chromosome is both independent from and synergistic with the effects of sex hormones. Loss of the Y chromosome is frequently observed in BC patients, while an extra copy of the X chromosome confers better protection against BC in females than in males. Advent of advanced technologies such as multiomics and artificial intelligence will likely further improve the understanding of sex differences in BC, which may ultimately lead to personalized preventative and treatment strategies depending on the biological sex of patients. This review delves into the impacts of biology of sex on BC, emphasizing the importance of further research into sex-specific biology to improve cancer prevention and care.

## Highlights

1. Male sex constitutes the most significant independent risk factor for bladder cancer.
2. Sex as a biological variable consists of canonical (i.e., sex chromosomes and sex hormones) and non-canonical (e.g., epigenome, immunity, microbiota and metabolism) sex-biasing factors.
3. Precision medicine must integrate sex-dependent differences to improve diagnosis, treatment, and outcomes.

## Abstract (plain language summary)

Cancer strikes everyone but the odds differ among individuals. For reasons that are only partially understood, men are much more likely than women to develop cancers. For instance, BC is four times more frequent in males than in females. The purpose of this article is to review recent advancement how biology of sex impacts cancers, focusing on BC. The article reviews the roles of canonical or known sex-biasing factors including male- and female-specific reproductive hormones and chromosomes, as well as emerging non-canonical sex-biasing factors such as immunity, metabolism, and commensal bacteria. We argue that a better understanding of these sex-biasing factors is needed to catalyze development of tailored strategies for men and women to improve cancer prevention, diagnosis, and therapy.

## Introduction

One of the most enigmatic observations of cancer epidemiology is that cancer at the shared anatomic sites strikes males more frequently than females. The pervasive nature of male predominance in cancers has been demonstrated across racial, geographic and social economic backgrounds, as well as in experimental cancer models, suggesting that biology of sex plays a central role in cancer. Bladder cancer (BC) is a prototypical sex-biased non-reproductive cancer type [1]. Incidence rate of BC is about four times higher in males than in females, and twice as high in white men compared to black, Hispanic, or Asian/pacific islander men [2]. In an experimental model of bladder carcinogenesis, male mice are over 13 times more likely than female mice to develop BC [3]. Worldwide, there were approximately 614,298 new cases and over 220,596 deaths of BC in 2022 [4, 5]. In the United States alone, there is an estimated 83,190 new cases (approximately 63,070 in men and 20,120 in women) and about 16,840 deaths from BC (about 12,290 in men and 4550 in women), comprising about 4% of all cancers in 2024 [2]. Majorities of BC are urothelial cancer originated from bladder urothelium, which accounts for over 90% of all cases. Recent findings have highlighted fundamental biological differences between male and female tumor cells and tumor microenvironment. However, advancement of basic research has not translated into any clinical practices as BC management remains largely the same for male and female patients.

## Histologic and molecular pathology of bladder cancer

UC is categorized into two main groups based on clinical staging: non-muscle invasive BC (NMIBC) and muscle invasive BC (MIBC) [6]. Approximately 80% of BC cases are diagnosed as NMIBC. NMIBC is often considered an early-stage BC where the cancer cells are confined to the bladder’s inner lining, or urothelium, and have not spread into the deeper muscle layers, while MIBC are high-grade tumors that invade the muscularis propria of the bladder, or beyond [6]. MIBC shows variable histological heterogeneity. There are multiple well established histologic subtypes of BC based on morphology (Table 1). Some of the histologic subtypes (micropapillary, plasmacytoid) have aggressive clinical courses compared to conventional BC [7]. Several approaches have been developed to define molecular subtypes of BC, evolving with 3 major molecular subtypes including luminal, basal, and neuroendocrine types [8, 9]. These molecular subtypes differ in molecular alterations, histomorphology and immune signatures. Luminal cancers harbor fibroblast growth factor receptor 3 (*FGFR3*) mutations and cyclin-dependent kinase inhibitor 2 A (*CDKN2 A*) losses whereas basal cancers are rich in tumor protein p53 (*TP53*) mutations and neuroendocrine cancers show concurrent mutations of *TP53* and retinoblastoma (*RB1*) as seen in small cell neuroendocrine carcinomas [10]. Most of the basal cancers are histologically squamous bladder carcinomas whereas luminal cancers show various morphologies such as conventional invasive UC, micropapillary, nested or plasmacytoid morphology [8, 10]. Most common genetic alteration across all histologic subtype, grades and stages are telomerase reverse transcriptase (*TERT*) promoter mutations [11–13]. This finding is helpful for distinguishing deceptively bland UC subtypes such as nested carcinoma from its benign mimics and for differentiating small cell neuroendocrine carcinoma of bladder origin versus lung origin. Detection of *TERT* mutation in urine specimens is a useful biomarker for early detection and monitoring of recurrence [13]. BC tumor microenvironment (TME) involves immune cells and tumor-associated stromal cells. The molecular signature of the TME differs amongst molecular and histologic subtypes and obscure meaningful differences among different BC subtypes. The Lund system excludes TME signatures from molecular subtypes [11]. Differences in clinical outcomes relate to TME signatures as well as cell-cycle activity. Studies have shown that immune checkpoint inhibitors (ICI) response is seen in 50% patients with high tumor mutation burden, Programmed cell death ligand-1 (PD-L1) expression, and infiltration by cytotoxic T-cells without significant difference among different molecular subtypes, questioning clinical utility of molecular subtypes [14, 15].

**Table 1:** Histologic subtypes of bladder cancer

Histologic subtype	Diagnostic criteria
Conventional urothelial carcinoma (UC)	Invasive carcinoma with evidence of urothelial origin
UC with squamous differentiation	Conventional UC with transition to squamous differentiation
UC with glandular differentiation	Conventional UC with transition to glandular
UC with trophoblastic differentiation	Conventional UC with syncytiotrophoblastic cells
Nested	Nested architecture
Micropapillary	Multiple small clusters within lacunar spaces
Plasmacytoid	Single, dispersed plasmacytoid cells
Tubular and microcystic	Cysts, macrocysts or large tubular structures
Lymphoepithelioma-like	Syncytial clusters of UC within a polymorphic inflammatory infiltrate
Lipid-rich	Cytoplasm with lipid vacuoles
Osteoclast-like giant cell rich	High grade UC with osteoclast-like giant cells
Sarcomatoid	UC with transition to spindle cell/sarcomatoid morphology
Poorly differentiated	Poorly differentiated morphology

The clinical utility of molecular subtyping for triage of patients for neoadjuvant chemotherapy has been explored. Study of Lotan et al., [16] showed that neoadjuvant platinum-based chemotherapy improves survival in patients with non-luminal subtypes of BC using Decipher assay. Contradictory results were shown in a study by Sjödahl et al., in which genomically unstable cancers disproportionately benefited from cisplatin-based neoadjuvant chemotherapy, using the Lund system [12]. These findings preclude interchangeability of different molecular systems used for molecular subtyping of BC but may help identify patients likely to respond to cisplatin based neoadjuvant chemotherapy.

## Bladder cancer management

The most common presenting symptom of BC is hematuria. Microscopic hematuria is associated with a 2.7% risk of harboring BC or other genitourinary malignancy [17]. Gross (visible) hematuria is associated with UC at a rate of up to 20% [18]. Most cases of microscopic hematuria and all cases of gross hematuria should prompt evaluation of the lower urinary tract via cystoscopy (most often flexible cystoscopy in the outpatient clinical setting) and evaluation of the upper urinary tract via imaging (most often intravenous contrast enhanced CT scan with delayed imaging, or CT Urography) [19]. A total of 78% of men directly consult a urologist, compared to only 55% of women [20]. Women are more often misdiagnosed with urinary tract infections (UTI) and less likely to undergo imaging [21], potentially driving the sex-based outcome disparities. Patients with a bladder mass suspicious for malignancy should be further managed with transurethral resection of bladder tumor (TURBT). This outpatient procedure under general anesthesia is performed for both diagnostic and therapeutic purposes. The pathology result at TURBT will convey histologic diagnosis, tumor grade, and tumor stage. At the time of TURBT, examination under anesthesia is often performed, as this also provides essential information regarding tumor stage. TURBT is therapeutic because many cases of NMIBC will not require surgical management beyond a complete TURBT, excepting recurrence.

**Fig. 1:**
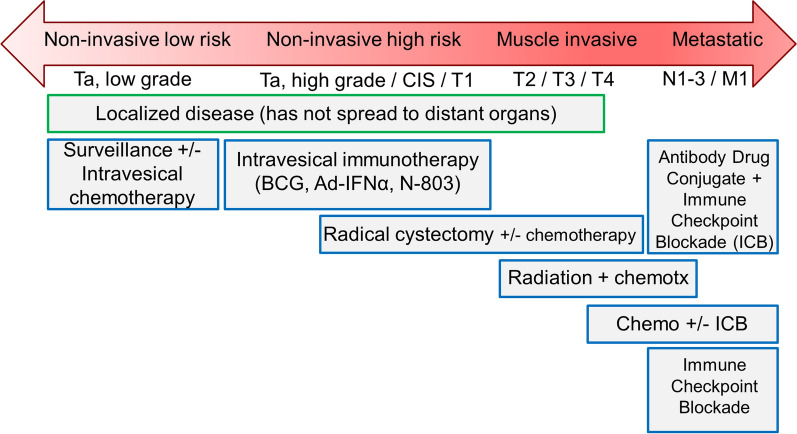
Bladder cancer grade and stage determine treatment options available to patients. Individual patient factors, preferences, and physician recommendations influence the final therapy choice. BCG, Bacillus-Calmette Guerin intravesical immunotherapy. Immunotherapy on the lower right refers to systemic treatments such as immune checkpoint inhibitors. The class of systemic bladder cancer drugs known as antibody drug conjugates can be considered a hybrid of systemic chemotherapy/immunotherapy approaches

Management options for BC vary based on tumor and patient factors [19, 22, 23]. Low-grade NMIBC tumors, papillary or finger-like growths that rarely invade deeper bladder tissues, can be managed with cystoscopic surveillance. Intermediate-risk NMIBC sometimes also include intravesical chemotherapy or immunotherapy [24]. High-grade NMIBC, such as carcinoma in situ (CIS), often requires repeated TURBT 4–6 weeks after the initial procedure, as well as intravesical immunotherapy with Bacille-Calmette Guerin (BCG) [25]. Treatment options for localized MIBC (clinical grades: N0 M0) include radical cystectomy [26] or trimodal bladder preserving therapy (radical/maximal TURBT, systemic chemotherapy, and pelvic radiation therapy) [27]. Radical cystectomy includes total removal of the bladder (and prostate in males and sometimes uterus, ovaries, and anterior vaginal wall in females), in addition to removal of the bilateral pelvic lymph nodes [28], followed by urinary diversion. Urinary diversion typically repurposes a segment of ileum to construct an ileal conduit or orthotopic neobladder. For patients undergoing radical cystectomy, neoadjuvant chemotherapy meaningfully improves overall survival [29]. Metastatic BC can be treated with systemic chemotherapy [30], systemic immunotherapies [31–33], and most recently, antibody drug conjugates [34, 35]. A visual summary of the range of treatment options for the spectrum of BC is shown in Fig. 1.

Common BC treatments pose specific toxicities and risks to patients. For example, intravesical BCG is associated with a 75% incidence of irritative lower urinary tract symptoms and 1–2% risk of serious or septic infection [36–40]. Radical cystectomy is associated with a 50–60% complication rate and 3–4% risk of 90-day mortality [41]. Systemic immunotherapies are typically better tolerated by patients than cytotoxic chemotherapies, yet intravenous ICI are associated with a 10–15% rate of serious adverse effects that include rash, pneumonitis, hypothyroidism, hepatitis, and colitis [31, 33].

Intravesical recurrence is a hallmark of NMIBC, with high rates of tumor return following initial treatment. In select cases of NMIBC treated with BCG immunotherapy, the risk of recurrence over 3 years decreases significantly, from approximately 40 to 75% [25]. MIBC are potentially curable, with 60–65% of patients undergoing radical cystectomy (+/−neoadjuvant chemotherapy) alive and disease free 5 years after treatment [26–29]. Metastatic BC are generally thought to be incurable, but the recent advance of enfortumab vedotin antibody drug conjugate combined with pembrolizumab immune checkpoint inhibition has tremendously increased median overall survival (32 months) compared to systemic chemotherapy (16 months) [35]. Although the incidence of BC is strongly male-biased, in patients with high stage disease, survival outcomes are worse in females [1, 42, 43], but the basis for this remains a debated issue [44].

## Risk factors of bladder cancer

### Biological sex

BC is associated with several risk factors, with sex as biological variable (SABV) playing a significant role in its incidence, progression, metastasis and treatment response [45–47]. Male sex consistently experiences an elevated risk in BC. Studies show that men are 2–4 times more likely to develop BC than women, even after adjusting for known risk factors like smoking, occupational exposures, and infections [48]. Beyond BC, sex differences extend to metastasis, immune responses, and treatment efficacy across multiple cancer types. Androgen signaling has been linked to worse cancer progression and therapy resistance, as seen in melanoma, where males exhibit impaired tumor control due to higher androgen receptor (AR) expression. Pharmacological inhibition of AR improves therapeutic response, emphasizing the critical role of sex hormones in cancer risk and treatment outcome [49]. In glioblastoma, tumor growth in males and females is driven by different pathways, with the cell cycle driving tumor progression in men and integrin signaling driving tumor progression in women, affecting chemotherapy response and underscoring the need for sex-specific treatment approaches [50]. These findings highlight the necessity of incorporating SABV in cancer research to develop more effective, sex-specific therapeutic strategies.

### Cigarette smoking

Cigarette smoking is a major behavioral risk factor for cancers in many tissues, including lung and bladder [2, 51]. Cigarette smoking introduces carcinogens like aromatic amines and *N*-nitroso compounds [51]. Current smokers face a two to four times higher risk of BC compared to non-smokers, with former smokers at a threefold increased risk [51]. Previous Studies from 1995 to 2006 estimated that tobacco use accounts for 50–65% of BC cases in men and 20–30% in women, reflecting higher smoking rates among men [52]. Recent findings suggest that the proportion of BC cases attributable to smoking has become comparable between men and women, nearing 50% for both sexes [51]. Smoking has been associated with poor outcomes in both NMIBC and MIBC cases after diagnosis [53]. Additionally, smoking is associated with poorer surgical outcomes, increased complications, reduced chemotherapy efficacy, and heightened recurrence risk [54–56]. Cigarettes contain over 60 known carcinogens including 4-ABP, 2-naphthylamine, and polycyclic aromatic hydrocarbons which are known BC-causing agents [57, 58]. These carcinogens may cause inflammation, oxidative stress, DNA damage, and mutation of oncogenes and/or tumor suppressor genes to promote carcinogenesis [58–60]. Electronic cigarette smoking also induces DNA damage and inhibits DNA repair in the mouse lungs and bladder urothelium and developed bladder urothelial hyperplasia, indicating a potential bladder carcinogen in mice [61]. In addition to the established genotoxic effect of cigarette smoking, it may increase BC risk by eliciting chronic albeit sub-clinical immune suppression [62].

Occupational and environmental exposures to chemicals, especially in industries such as aluminum production, rubber manufacturing, dye and textile production, coal-tar pitch, and dry cleaning, further elevates risk through carcinogens like 2-naphthylamine, benzidine, 4-aminobiphenyl (4-ABP), polyaromatic hydrocarbons, 4,4′-methylene-bis(2-chloroaniline), and tetrachloroethylene [63]. Environmental exposures, including arsenic in drinking water, also significantly increase risk [64]. Furthermore, gene-environment interactions play a crucial role, where genetic polymorphisms in detoxification genes, notably N-acetyltransferase 2 (*NAT2*) and Glutathione S-transferase Mu 1 (*GSTM1*) increases the risk of BC [65]. Individuals with GSTM1 null genotype had a significantly increased the overall risk of BC, and the *NAT2* slow acetylator genotype increases risk particularly among cigarette smokers [65].

### Infection

*Schistosoma haematobium* infection is another know risk factor of BC, and the World Health Organization classifies *Schistosoma haematobium* parasite as a group 1 human carcinogen [66]. BC from *Schistosoma haematobium* likely arises due to chronic inflammation and DNA damage from immune responses triggered by the parasite’s eggs lodged in bladder tissue. *Schistosoma haematobium* infects humans by directly penetrating the skin through aquatic cercariae, which emerge from *Bulinus truncatus*, the parasite’s intermediate snail host. Upon entering the human body, the parasite quickly migrates into the bloodstream in its *schistosomulae* form, matures, and eventually settles in the venous plexus of the bladder. There, male and female worms pair, mate, and produce eggs, continuing this cycle [67]. The half of those eggs are excreted in the urine. The remaining eggs become trapped in the bladder wall, ureters, and genital tract. The deposition of eggs triggers chronic inflammation, releasing of growth factors and other biochemical substances such as *N*-nitroso compounds with carcinogenic effects and weakening the local immune system, allowing co-infections that promote cancer [66, 68].

Unlike the parasite infection, the impact of UTI caused by uropathogenic bacteria on BC risk is less clear. BC shares several clinical symptoms with UTI, including painful urination, urgency, frequent urination, and hematuria [21, 69–75]. The study conducted by Vermeulen et al., reported in regular, low-grade UTI are associated with an elevated risk of BC, particularly in MIBC, with stronger effects observed in men (OR 6.6) than in women (OR 2.7). Interestingly, however, a smaller number (fewer than 5) of UTI episodes treated with antibiotics in younger individuals seems to reduce BC risk. Additionally, postmenopausal UTI significantly raise the risk regardless of the number of infection episodes [76, 77]. Chronic or recurrent UTI can lead to persistent inflammation and damage to the bladder lining [78], potentially increasing the chances of mutation, which may increase the BC risk [79].

## Molecular basis of sex differences in bladder cancer

**Fig. 2:**
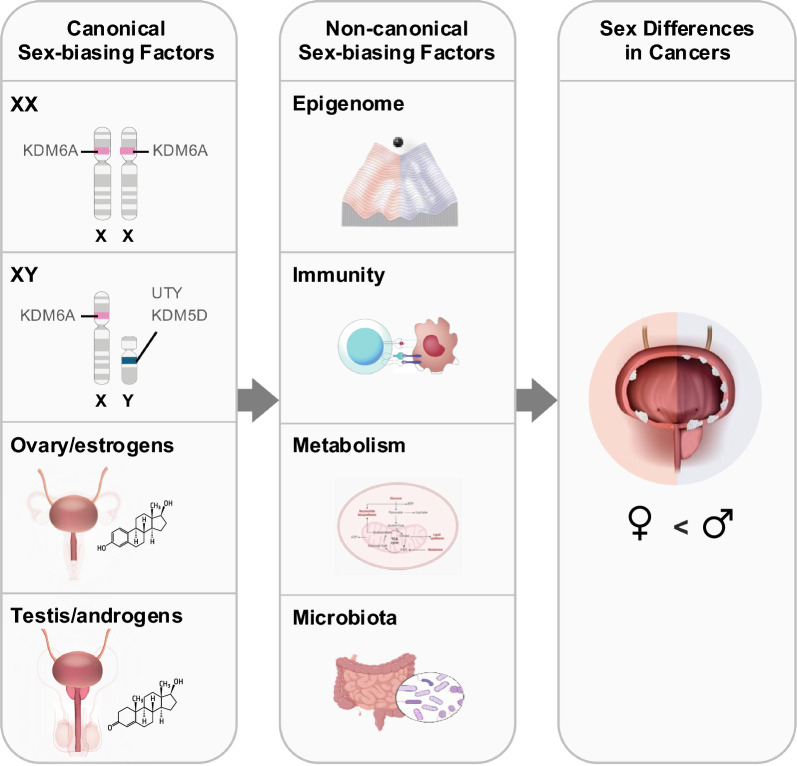
The impact of the canonical and non-canonical sex-biasing factors on bladder cancer. This diagram illustrates how canonical factors (sex chromosomes and hormones) and non-canonical factors (epigenetic modifications, immune response, metabolism, and microbiota) contribute to the observed sex bias in bladder cancer

Sex, a biological variable encoded in DNA, fundamentally shapes many aspects of development and diseases. In humans and other mammals, effects of the Y chromosome-encoded transcription factor Sex-determining Region Y (SRY) instigate formation of the testes over ovaries, linking the chromosomal sex (XX vs. XY) to gonadal sex (ovary vs. testis) and hormonal milieu (estrogens vs. androgens) (Fig. 2). These generally aligned canonical sex-biasing variables, i.e., sex chromosomes, gonadal types, and sex hormones, are confounding variables, where effect of one may obscure or alter the effects of another. The canonical sex-biasing factors may directly contribute to cancer development. They may also indirectly impact cancer via other non-canonical sex-biasing factors, such as the epigenome, immunity, metabolism, and microbiome (Fig. 2). Together, sex-biasing effects of these biological variables may complement or amplify each other’s impacts on cancers [45].

### Sex hormones

Sex hormones, including androgens and estrogens, play a crucial role in shaping the physiological and structural differences between males and females [80]. Their impact goes well beyond reproductive functions, as these hormones profoundly influence various physiology and pathophysiology, including cancers [81]. Research on sex steroid hormones and their receptors in BC has spanned nearly 50 years by various groups.

Androgens are a class of sex hormones predominantly synthesized by the testes, ovaries, and adrenal cortex, with elevated levels observed in males. These hormones are essential for the development of the male reproductive system [82]. The role of androgens and AR signaling in the pathogenesis of prostate cancer is well-documented [83, 84]. Binding of testosterone, the most prevalent androgen, to the ligand-binding domain of the AR (NR3 C4; nuclear receptor subfamily 3, group C, member 4) induces a structural change that facilitates nuclear translocation of the receptor: ligand complex, where it initiates the transcription of a variety of target genes [84]. In 1972, androgens were first identified as a BC risk factor when castrated male mice were better protected against N-butyl-N-(4-hydroxybutyl) nitrosamine (BBN) than uncastrated males, and female mice treated with testosterone had increased BC risk compared to control female mice [85]. Further study also suggest that androgens promote BC development, while estrogens help to prevent it [86]. Additionally, clinical observations show that men who take drugs blocking androgens, like 5α-reductase inhibitors or DHT blockers, have lower BC death rates. This highlights the role of androgens in BC [87]. The next breakthrough came with the characterization of the AR and androgen-AR signaling. Laor et al., showed that AR levels are higher in bladder tumors compared to normal tissue and are also higher in males than in females [88]. Mice with AR gene deletions (germline or urothelium-specific) show reduced susceptibility to BC when exposed to a bladder-specific carcinogen [89, 90]. Consistently, overexpression of AR increases the susceptibility of BC during carcinogen exposure [91]. Genetic studies strongly showed that the AR plays a critical role in promoting BC. To understand whether urothelial AR (Uro-AR) has any role in promoting bladder tumorigenesis, Hsu et al., generated conditional Uro-ARKO mice (Uro-AR−/y) that lacked AR only in urothelium. They found that Uro-AR could promote bladder tumorigenesis by modulating the p21 protein—a key regulator of the cell cycle and DNA repair through the p53-PCNA signaling pathway [90]. However, Miyamoto et al., demonstrated, AR and androgens do not always need each other to drive cancer growth. For instance, male mice without AR that were treated with the hormone DHT still developed BC 25% of the time, while untreated mice without AR did not develop cancer [92]. This suggests that androgens can promote BC through a pathway that does not involve AR. A recent study shows that DHT promotes BC cell proliferation and invasion via EPPK1-mediated mitogen-activated protein kinase (MAPK)/junction plakoglobin (JUP) signaling rather than AR [93]. Chen et al., identified a novel membrane AR (mAR-SLC39 A9) that promotes BC through a noncanonical AR pathway involving Gαi/MAPK/MMP9 [94]. They also show that Cd24a-deficient C57BL/6 male mice develop fewer BBN-induced bladder tumors than untreated males. In addition to this, Cd24a-deficient male mice also had fewer metastases than wild-type counterparts. More interestingly, AR knockdown in UM-UC-3 and TCCSUP human UC cell lines resulted in suppression of CD24 expression and cell proliferation, and androgen treatment also led to increased CD24 promoter activity, dependent on the presence of androgen receptor. In vivo, androgen deprivation resulted in reduced growth and CD24 expression of UM-UC-3 xenografts, and the latter was rescued by exogenous CD24 overexpression [95]. Intriguingly, AR directly suppresses the transcription of CD44, a receptor for hyaluronic acid and a strong marker for aggressive disease in various tumors [96]. This suggests that AR might play different roles depending on the stage of BC, potentially promoting tumor initiation but inhibiting progression.

A recent prospective study and meta-analysis have concluded that there is a greater risk of BC among nulliparous women and among women with early menopause, suggesting the protective effect of female sex hormones [97]. Preclinical models showed that ovariectomy increased the incidence of BBN-induced bladder tumors, compared to controls [86], while 17β-estradiol treatment reduced tumor formation [98], suggesting the preventive effects of estrogens on UC growth. Estrogens primarily interacted with two canonical nuclear receptors known as ERα and ERβ. Each receptor can have unique roles, which may vary depending on the specific tissue type involved [99, 100]. Differential expression of ERα and ERβ in human BC suggests distinct roles in tumor development, with ERα likely inhibiting initiation and invasion [101] and ERβ promoting initiation and progression [102]. Hsu et al., demonstrated that female mice lacking ERα develop tumors faster than those with functionally active ERα, indicating a possible tumor-suppressive role for ERα in cancer development [103]. However, ERβ shows increased expression in advanced-stage and higher-grade BC, implying a role in tumor progression and metastasis. Indeed, deletion of ERβ led to reduction of tumor growth [104]; and Tamoxifen, a selective estrogen receptor modulator (SERM) that inhibits ER activity, provides chemoprevention against urothelial carcinogenesis in mice [105]. Estrogen signaling is also mediated by the membrane-bound G protein-coupled estrogen receptor (GPER). GPER is involved in the rapid non-genomic actions of estrogen, involving downstream signaling pathways by inducing epidermal growth factor receptor (EGFR), MAPK, protein kinase A (PKA), and phosphoinositide 3-kinase (PI3 K) pathways [106]. Activation of GPER by estrogen suppressed bladder urothelial cell proliferation [107]. In addition, GPER is associated with the immunoregulatory function of estrogen. It has been shown that GPER is expressed in multiple immune cells, regulating their activation and life span. These include neutrophils, monocytes/macrophages, B and T lymphocytes, as well as eosinophils, and neutrophils [108]. GPER may therefore modulate the TME that impacts tumor progression [108]. Collectively, estrogen plays a complex role in bladder tumor development via both the intrinsic and extrinsic mechanisms.

Progesterone (P4) is an endogenous steroid sex hormone secreted by the ovary which regulates female reproductive functions [109]. P4 actions are mediated by two nuclear progesterone receptors (PR), progesterone receptor A (PR-A) and progesterone receptor B (PR-B) [110], which are transcribed from a single gene but from different promoters in response to estrogen. PR-B has an extra 164 amino acids at the N-terminus [111]. Non-genomic actions of P4 are mediated by G protein-coupled PR on cell membranes [112]. PR expression is used as a biomarker of ER-α function. It has been shown that PRs are not only an ER-α-induced gene target but is also an ER-α-associated protein that modulates its activity [113]. Johnson and his coworker, using the UPII-SV40 transgenic model in which BC spontaneously develops, found that tumor size was significantly smaller in multiparous female mice than in nulliparous ones [114]. Consistently, it was also reported in humans that multiple pregnancies are associated with a decreased risk of BC [115]. It would be interesting to find out if progesterone acts on its own or works with the estrogen receptor (ER) to increase BC risk in women.

### Sex chromosomes

In mammals, males and females have different sets of sex chromosomes: males have one X and one Y chromosome, while females have two X chromosomes. The Y chromosome encodes for a specific region known as “Sex-determining Region Y” (Sry), which triggers the development of the testes. The testes then release androgens that lead to male-specific physical traits. Historically, we have focused more on the role of sex hormones, with less attention given to the potential impact of sex chromosomes in explaining differences in BC between males and females. Emerging evidence suggests that the sex chromosomes directly contribute to differences in male and female BC biology independent from the effects of sex hormones [3, 116, 117]. A 2008 epidemiological study revealed that individuals with Turner syndrome, phenotypic females who completely or partially lack one X chromosome (XO), had a higher risk of BC compared to XX females [118]. Furthermore, patients with Klinefelter syndrome, phenotypic males with one or more extra X chromosomes (e.g., XXY), exhibited a lower risk of solid tumors than XY males [119]. While these studies pointed to possible roles of sex chromosomes in cancer risk, they were largely correlative and did not isolate the independent effects of the X and Y chromosomes, as confounding factors like chronic UTI, fluctuating sex hormone levels, and other pre-existing health conditions that cannot be controlled for. To investigate the role of sex chromosomes (XX vs. XY) in BC risk, we employed age-matched “four-core genotype (FCG)” mice [3, 116], comprising four distinct sex types: two with testes (either XX or XY chromosomes) and two with ovaries (either XX or XY chromosomes) [120]. This FCG model effectively separates the sex chromosome effect (SCE) from gonadal hormone effect (GHE), allowing for precise evaluation of both independent and interactive activities of these canonical sex-biased factors [120]. We administered BBN to the FCG mice and tracked BC development and overall survival outcomes. We found the Y chromosome does not confer the same level of protective effects against BC as the X chromosome does [3]. Moreover, cox proportional analysis has confirmed that the testis or androgen hormones independently contribute to sex-biased effects, with a hazard ratio of 4.714 (95% CI = 2.77–8.28). Additionally, the analysis revealed that the sex chromosome complements acts as a sex-biased risk factor independent of gonadal hormones, with a hazard ratio of 2.549 (95% CI = 1.55–4.28). Interestingly, an interaction was observed between gonadal hormone effects (GHE) and sex chromosome effects (SCE), as the combined hazard ratio for both factors was 12.39 (95% CI = 5.54–31.63), consistent with the product rather than the combination hazard ratio of SCE and GHE. These results suggest, for the first time, that SCE and GHE may interact synergistically to enhance sex differences in BC.

As we age, DNA damage naturally builds up in our cells, but our bodies have built-in tumor suppressor systems to keep tissues functioning normally and protect us from cancer. Interestingly, cancer rates increase faster with age in men than in women [121, 122]. One possible reason for this difference could be the protective role of the X chromosome in women. The X chromosome carries several important genes that act as tumor suppressors, epigenetic regulators, and interactors with the p53 pathway [123, 124]. Women, who have two X chromosomes, have a backup for these genes, which can help protect against mutations in one of the X chromosomes. About 15% of genes on the X chromosome escape a process called “X-inactivation,” meaning they stay active on both X chromosomes [125]. Some of these genes are involved in tumor suppression. If one X chromosome in a woman has a mutation in these genes, the second X chromosome can still provide a functional copy, which is protective. Men, with only one X chromosome, don’t have this backup, making them more vulnerable to mutations in these genes. Certain genes like Lysine demethylase 6 A (KDM6 A) also known as *UTX* (ubiquitously transcribed tetratricopeptide repeat on the X chromosome), *ATRX* (alpha-thalassemia mental retardation X-linked), and *DDX3X* (DEAD-box helicase 3 X-linked), which play roles in preventing tumors, are often more mutated in male cancers [126]. KDM6 A likely has both catalytic and non-catalytic mechanisms through which it modulates the epigenetic landscape, and both activities appear to be important in its tumor suppressor activity through downstream effectors such as CDKN1 A and PERP [3].

It was reported that in men over 70, > 0% have a detectable loss of the Y chromosome, a condition known as mosaic loss of the Y chromosome (mLOY) [127]. mLOY can disrupt normal cellular functions, including cell cycle regulation, DNA repair, and apoptosis. These disruptions can contribute to cancer development and progression. Studies have shown that mLOY leukocytes are associated with an increased risk of cancer-related mortality [128]. mLOY might originate in cells with TP53 mutations and highly aneuploid tumors which are associated with genomic instability, however, in some cancers, mLOY does not always result from genomic instability [129]. It was shown that mLOY could contribute to the genomic instability using a murine model of BC [117]. mLOY could promote immune evasion by disrupting the T cell function and upregulating the expression of immune checkpoint molecules, such as CD274, LAG3, and HAVCR2, resulting in T cell exhaustion and increased susceptibility to PD-1-targeted immunotherapy which is an essential treatment for BC [117, 130]. The Y chromosome encodes few genes, and these genes are mostly expressed in the reproductive tissues. However, some of these genes are also expressed in nonreproductive tissues including cancer cells [131]. Two of the Y chromosome genes, KDM5D and UTY are associated with tumor suppression. KDM5D inhibited growth and progression of prostate and gastric cancers by demethylating H3 K4 me3 leading to the suppression of matrix metalloproteinases expression [132, 133]. It has been reported that UTY deficiency can promote the development and progression of BC [117]. Mosaic LOY in leukocytes being associated with increased cancer incidence suggests the possibility that mLOY regulates immunosurveillance and cancer cell-intrinsic biology [134]. It has been suggested that the association between LOY and disease risk depends on what type of leukocyte is affected with Y loss, with prostate cancer patients showing higher levels of mLOY in CD4^+^ T cells. mLOY in regulatory T cells (Tregs) influences their ability to regulate immune responses, leading to cancer progression by altering the TME and immunosurveillance [135, 136]. Just like cancer cells are more sensitive to chemotherapy than normal cells, we speculate that a patient’s nonmalignant blood cells with mLOY are more affected by cancer treatment compared to cells without mLOY. Taken together, this emerging field of Y cancer biology is an exciting area requiring further investigation to uncover the role and function of cells with LOY and the potential interactions between mLOY cancer cells and mLOY tumor-infiltrating immune cells within the context of cancer progression. Furthermore, LOY or specific Y-linked gene alterations when combined with yet to be detected features may be found to be novel and powerful biomarkers for cancer risk assessment and early detection in men. Understanding the role of Y chromosome genes in cancer may also lead to the development of targeted therapies whose specificity is related to genetic and molecular abnormalities associated with the Y chromosome.

### Sex epigenome

Epigenetic regulation is crucial for genetic modulation and drives phenotypic diversity. The “sex epigenome” refers to sex-specific differences in gene regulation, post-translations modifications and chromatin organizations that are modulated by canonical sex-biasing factors such as sex hormones and sex chromosomes. Epigenetic modifications such as methylation and acetylation on DNA and histones to control gene activity, either silencing or activating specific genes. This enables them to respond to biological and environmental changes. The sex epigenome theory posits that through time effects of the canonical sex-biasing factors result in the sex-specific epigenetic landscape, which independently contribute to sex differences in health and diseases. In cancer, the epigenome plays a key role, with epigenetic regulators and those regulators are frequently mutated in BC compared to other solid tumors [137, 138]. Hypomethylation is typically linked to disease progression [139, 140]. For instance, hypomethylation of long interspersed nuclear elements-1 (LINE1) in peripheral blood-derived DNA has been associated with an increased risk of BC, particularly among female patients, who exhibit significantly higher rates compared to males [141]. This LINE1 hypomethylation increases oxidative stress, promoting tumor progression, and can induce an alternate splice variant of the MET oncogene in bladder tumors [142, 143]. Therefore, LINE1 hypomethylation contribute to the higher incidence of high-grade disease in female BC patients. Conversely, male patients more commonly exhibit DNA methylation of the DNA topoisomerase 2 beta (TOP2B) gene [144], although its role in BC remains unclear.

In the BBN treated mouse model of BC, XY males are 12.39 times more likely than XX females to develop and die from BC. Our study has demonstrated, urothelium-specific deletion of KDM6 A significantly elevated BC risk of females [3]. Moreover, we observed the deleting KDM6 A can significantly lowered the male-to-female BC risk ratio by over five times. This finding also suggests that KDM6 A acts as a tumor suppressor through both demethylase-dependent and demethylase-independent mechanisms and show a stronger protective role against BC in females. Our data has shown, KDM6 A promoted expression of known canonical TP53 gene targets such as CDKN1 A and PERP [3, 145]. Conversely, KDM6 A conditional knockout male mice did not have worse survival compared to their female counterparts. This may be due to the Y chromosome encoded UTY, a paralog of KDM6 A. UTY has been shown to have no or minimal demethylase activity [146, 147]. Nevertheless, UTY may compensate the loss or mutation of KDM6 A on the X chromosome in males. Polycomb repressive complex 2 (PRC2) is involved in compacting the chromatin and controlling gene expression. In bladder urothelium, enhancer of zeste homolog 2 (EZH2), the methyltransferase component of PRC2, works in opposition to KDM6 A, catalyzing addition of methyl groups to H3 K27 residues [148]. Since KDM6 A mutations are more common in women with NMI BC, future research should explore the use of EZH2 inhibitors to target KDM6 A-related pathways in female BC patients [149]. In conjunction with its demethylase role of KDM6 A, it interacts with COMPASS, a protein complex family that includes MLL3/KMT2 C and MLL4/KMT2D [150]. Current evidence suggests that KDM6 A reduces tumor formation through two histone-modifying mechanisms: (1) counteracting PRC2-dependent gene repression by blocking H3 K27 tri-methylation, and (2) supporting COMPASS-dependent gene activation by enabling H3 K4 mono-methylation [151]. While the specific roles of PRC2 and COMPASS in influencing sex differences in BC are not yet confirmed, their varied and essential functions in development and cancer highlight their potential in addressing sex-related differences in BC development.

### CD8^+^ and CD4^+^ T cells

CD8^+^ T cells, also known as cytotoxic T lymphocytes, are an important part of the adaptive immune system and are crucial components of the anti-tumor immune response. Through their T cell receptor, they can recognize tumor antigens and exert their cytolytic activity over their target cells [152]. In states of continuous antigen stimulation, such as cancer, the CD8^+^ T cells progress into a state known as T cell exhaustion, in which the expression of inhibitory receptors on their surface attenuates the cytotoxic function [153, 154]. A growing body of literature has recently begun to uncover how CD8^+^ T cells contribute to sex disparities in bladder and other types of cancer [155]. The role of sex hormones, particularly androgens, as well as genetic factors, such as LOY, have emerged as important modulators of CD8^+^ T cell mediated immune responses that influence cancer progression and response to therapy [156]. One of the intriguing aspects of CD8^+^ T cell function in cancer derives from the fact that AR is expressed not only in prostate tissues but also in specific subsets of CD8^+^ T cells [116, 157, 158]. Androgen activity has been predicted to orchestrate a complex transcriptional network involving several transcription factors that shape the differentiation and function of CD8^+^ T cells in a male- or female-biased manner [116]. In the context of BC, it has been shown that androgen-mediated AR activity can promote T cell exhaustion, a state manifested with increased expression of inhibitory receptors on CD8^+^ T cells and decreased CD8^+^ T cell effector function [116]. Mechanistically, androgens have been found to bind directly to androgen response elements on the promoter of the transcription factor Tcf7 to positively regulate its transcription [116]. This is believed to account for the higher abundance of Tcf7^+^ progenitor exhausted CD8^+^ T cells in the TME in male mouse models of BC [116]. Other possible mechanisms of androgen mediated CD8^+^ T cell suppression have been also reported, although not yet shown in BC [159]. Furthermore, LOY, a common genetic alteration in male BC patients, was recently reported to have a profound effect on CD8^+^ T cell mediated antitumor responses [117]. By employing a syngeneic mouse model of MB49 murine bladder tumors where all cancer cells exhibit complete LOY, it was demonstrated that LOY enhances tumor growth in a T cell dependent manner, mainly through accumulation of exhausted CD8^+^ T cells within the tumor microenvironment. Interestingly, in the same preclinical studies, BC models with LOY responded better to immune checkpoint blockade (ICB) therapy [117]. The role of CD8^+^ T cell intrinsic LOY in BC has not been fully studied yet. Other sex related mechanisms that might be associated with altered CD8 T cell function include tumor mutational burden and neoantigen load [160].These findings underscore the clinical relevance of the sex-specific differences in CD8^+^ T cell immunity, as a pathway to developing personalized and more effective treatment strategies for BC:

1. Targeting the androgen signaling pathway: therapeutic strategies that attenuating androgen signaling could enhance CD8^+^ T cell activity and improve anti-tumor immunity. In preclinical models of BC, it has been shown that surgical or pharmacological castration can improve the response to ICB [116].
2. Utilizing biological sex or LOY as a biomarker: although some with conflicting results, there are studies showing that male patients respond better to ICB therapy in various tumor settings [161–163]. Furthermore, LOY has also emerged as a potential biomarker for predicting responses to ICB therapies; LOY in tumor cells has been correlated with improved responses to anti-PD-L1 therapy in BC patients [117], suggesting its potential utility in guiding treatment decisions.

Sex differences in CD8^+^ T cell immunity in BC is mainly known to be influenced by androgen signaling and genetic alterations like LOY, which contribute to the unique immune landscapes observed between males and females and affect disease progression and response to therapy. Advancing our understanding of these mechanisms will be key to developing targeted therapies that improve outcomes for BC patients.

Cytotoxic CD4^+^ T cells are also gaining recognition as important players in anti-tumor immunity [164]. To understand the role of CD4^+^ T in BC, Oh et al., profiled CD8^+^ and CD4^+^ T cells from bladder tumors and adjacent tissues using single-cell RNAseq and TCRseq [165]. They observed minimal differences in CD8^+^ T cells across tumor and nonmalignant tissue, while CD4^+^ T cells showed unique cytotoxic and regulatory states within tumors. These CD4^+^ T cells can eliminate autologous tumors through an major histocompatibility complex (MHC) II-dependent mechanism [148]. Within tumors, multiple subsets of CD4^+^ T cells with cytotoxic functions have been identified, specifically the CD4GZMB and CD4GZMK populations. The CD4GZMB subset is characterized by expression of cytotoxic molecules such as granzyme B (Gzm B), perforin, granulysin (Gnly), and natural killer cell granule protein 7 (NKG7). In contrast, the CD4GZMK subset expresses high levels of granzyme K (Gzm K) and lower levels of NKG7. Both cytotoxic subsets secrete substantial amounts of the anti-tumor cytokines interferon-gamma (IFN-γ) and tumor necrosis factor-alpha (TNFα), contributing to their tumor-suppressive functions. These cytotoxic CD4^+^ T cells are capable of directly lysing tumor cells by recognizing antigens presented onMHC class II molecules expressed by tumor cells. However, their anti-tumor effects can be counteracted by CD4^+^ Tregs present within the tumor microenvironment, which may inhibit their cytotoxic function [165]. Research suggests a sex bias in CD4^+^ T cell-mediated immunity, with females typically exhibiting a higher proportion of CD4^+^ T cells than males, resulting in a more robust cell-mediated immune response [166]. This disparity is largely influenced by sex hormones, as estrogen tends to enhance immune function in females, while androgens have a suppressive effect in males [167]. However, the sex-biased effects of CD4^+^ T cell-mediated immunity in BC remain largely unexplored, indicating a potential area for further investigation.

### Myeloid cells and myeloid-derived suppressor cells

Myeloid cells in tumors include resident tissue macrophages as well as infiltrating bone marrow-derived neutrophils, monocytes and dendritic cells, which can have a variety of pro- and anti-tumor functions [168, 169]. Tumors can influence myeloid cell differentiation in the bone marrow and thus program myeloid cells to support their growth before they reach the tumor or exploit them to facilitate metastatic spread. Indeed, recent studies have shed new light on the origins of functionally distinct subsets of monocytes and neutrophils in the bone marrow [170, 171], and implicated distinct differentiation pathways in the production of monocyte and neutrophil subsets with pro- or anti-tumor properties [172, 173]. Sex differences and aging-associated changes can impact of all these processes. Macrophages have been reported to be the most sexually dimorphic immune cells, especially in their expression of type I interferon-stimulated genes (ISG) [174], which may promote anti-tumor immunity. Notably, the single strand RNA (ssRNA) receptor TLR7 is encoded on the X chromosome and can escape X inactivation in female cells, resulting in higher TLR7 expression [175]. Altered myeloid cell function during aging also likely contributes to cancer risk and treatment responses.

Myeloid-derived suppressor cells (MDSCs) are one of the major immune cell types that contribute to tumor-induced immune suppression and escape from immune elimination [172, 176]. MDSCs can be broadly divided into two main populations, polymorphonuclear- (PMN, also known as granulocytic, G−) and monocytic (M−) MDSCs [172, 176]. MDSCs exert potent immunosuppressive activities towards T cells and NK cells through a variety of mechanisms, including removal of arginine in the TME via expression of arginase 1 (Arg1), production of reactive oxygen species, reactive nitrogen species and adenosine [172, 176]. MDSCs have been found in a variety of human solid tumors [177, 178] including BC, and increased MDSCs levels in BC patients correlate with advanced disease stage and poor prognosis [179, 180]. Importantly, emerging evidence suggest that MDSCs contribute to resistance to anti-CTLA-4 and anti-PD-1/L1 blockade [181]. High frequency of circulating MDSCs was found to be associated with poor responses to immune checkpoint therapies in melanoma [182], breast cancer [183] and prostate cancer [184]. Thus, targeting MDSCs represents an attractive approach to modulate tumor immunity for treating cancers and improve immune checkpoint blockade therapies [172, 176]. The contribution of MDSCs to sex differences in BC has not been well defined. However, sex differences in MDSCs accumulation and function have been recently observed in other cancers [185].

### Inflammatory cytokines

Inflammatory cytokines are important mediators of immunity and regulators of TME [186]. Expression of several cytokines is upregulated in tumor tissues in comparison with normal tissues in many cancers, including BC [187]. Cancer cells can further manipulate cytokine-inducing pathways such as CD14/TLR receptor pathway to increase cytokine production and benefit from pro-tumorigenic signals [188]. Non-targeted inhibition of chronic inflammation by non-steroidal anti-inflammatory drugs targeting COX2 enzyme reduces BC risk and facilitates cancer cell death, indicating overall tumor-promoting role of inflammation within the BC microenvironment [189]. Levels of cytokines may also serve as biomarkers of prognosis and therapy resistance. Expression of several cytokines, such as IL-6, IL-1 or IL-10 can be further regulated in sex-dependent manner including by AR or ER signaling and transcription complexes such as selective AR modulators (SARM) and SERM [190, 191]. Overall, particularly due to patterns of various TLR expression, type I interferons and type 2 cytokines (IL-4; IL-10) have female sex bias, while the induction of IL-6 and other TLR2/4 dependent cytokines has a male bias [192]. Furthermore, cytokines may play a role during tumor development and progression or be induced and act only in a context of therapy, for example chemotherapies, immunotherapies, or BC specific therapy with BCG. Interestingly, BCG therapy, which is often effective, nevertheless induces production of IL-6 and IL-8, which are typically pro-tumorigenic [193, 194]. IL-4 and IL-10 represent “type 2” and immunoregulatory cytokines which correlates with poor prognosis and were mechanistically implicated into the development of various cancers. However, for BC the data is mostly correlational with these cytokines overproduced in tumor tissue and their potential correlation with disease relapse and progression [195, 196]. TGFβ pathway is instrumental for growth suppression of epithelial cells as well as for the establishment of immunosuppressive tissue and tumor microenvironment. Mutations in *TGFβR* genes as well as other components of TGFβ signaling pathway which render cells insensitive to the inhibitory effects of TGFβ are common in BC [197]. IL-6 promotes BC via activation of proto-oncogenic STAT3 [198] and also regulates invasive properties of BC cells [199], presumably by activating pro-survival and invasive gene expression program controlled by STAT3 in a context of various cancer. “Proof of principle” studies demonstrated feasibility of pharmacological neutralization of IL-6 pathway resulting in decreased BC growth [200]. The role of IL-17 in BC has also been established where inhibition of IL-17 further affected IL-6-STAT3 pathway and reduced tumorigenicity [201]. Interestingly, IL-17 is involved into resolution of chronic UTI [202] in a sex biased manner: its levels are higher in females and its production is repressed by testosterone. IL-17 therefore promotes resolution of infections and may be essential in preventing tumor initiating chronic inflammation. Interferons in general play robust role in anti-tumor defenses, regulating both proliferation and sensitivity to cell death for cancer cells, and boosting anti-tumor immunity; participating in tumor surveillance and tumor immunoediting [203]. This sparked a considerable interest in these cytokines not only as a key mechanistic anti-tumorigenic entity, but also as a potential therapy. Direct therapeutic usage of interferons in cancer produced mixed results largely due to their systemic toxicity and side effects. Type I interferons IFN-α and IFN-β suppress BC via induction of innate and adaptive immune responses and may have additional direct anti-tumor activities [204, 205]. Enforced expression of these cytokines either via viral delivery or potential activation of endogenous production was suggested as a potential therapeutic option in BCG-unresponsive BC [204, 205]. Type III interferons such as IFN-λ regulate macrophages in TME and inhibit BC progression [206] and may be considered as anti-cancer agents when delivered intratumorally. IFN-γ is a key effector cytokines for anti-tumorigenic NK, CD8 and CD4 T cells affecting cancer cells and multiple immune cells [207] and its proper induction and signaling is essential for the success of immunotherapies in many cancers and is required for suppression of BC [208]. IFN-γ levels also correlate with the response to therapies and beneficial prognosis in BC patients [209] and its signaling within the epithelial [210] and endothelial compartment is essential for the success of immune checkpoint immunotherapy in BC [211].

### Microbiota

The microbiota plays a significant role in cancers, impacting tumor development through immune modulation, biochemical interactions, and effects on cell proliferation and death. Research suggests that over one-fifth of malignant tumors may involve microbiota influences [212]. Recent research has highlighted a link between BC and alterations in the urinary microbiome [213, 214]. Females are more susceptible to UTI. Xu et al., found that individuals with BC showed a significantly altered urinary microbiota, with decreases in *Serratia*, *Proteus*, and *Roseomonas* and increases in *Acinetobacter*, *Anaerococcus*, and *Sphingobacterium* [215]. Studies indicate that urinary microbiota dysbiosis may influence BC progression [216, 217], suggesting that different microorganisms in male and female urine create distinct local environments that either support or hinder tumor formation. There appears to be a close interaction between sex hormones, the immune system, and the urinary microbiome [218]. Sequencing has revealed that *Lactobacillales* and *Corynebacterium* dominate the urinary microbiota in females and males, respectively. *Lactobacillales* protect against UTI, and oral administering *Lactobacillus casei* can delay BC recurrence [219]. Pederzoli et al., found that women with BC had higher *Klebsiella* levels than healthy women [220], likely due to DNA-damaging toxins released by *Klebsiella* [221]. These findings underscore that sex differences in urinary microbiota may contribute to sex disparities in BC risk. In addition to urinary microbiota, gut microbiota also plays a crucial role in BC. A recent Mendelian randomization study suggests a causal link between gut microbiota and urological cancers, identifying specific bacterial traits associated with bladder, prostate, and kidney cancer risks. Notably, *Bifidobacterium*, *Actinobacteria*, and the *Ruminococcus torques* group were linked to higher BC risk, while *Allisonella* was associated with a reduced risk of bladder and prostate cancer. These findings underscore the potential role of gut microbiota in influencing BC risk [222]. Study has demonstrated gut microbiota plays a significant role in chemical-induced bladder carcinogenesis through its metabolism of carcinogens. Using a mouse model exposed to BBN, researchers investigated how gut microbiota influences BBN-induced BC and its toxicokinetics. Mice treated with antibiotics to reduce gut bacterial load showed a 99.99% reduction in bacterial colonies and a significant decrease in BC incidence compared to untreated mice. Antibiotic treatment reduced BCPN (an oxidized BBN metabolite) levels in bladder tissues, suggesting that microbiota promotes BBN metabolism to carcinogenic intermediates [223]. Furthermore, analysis of BBN-metabolizing bacteria identified *Escherichia*, *Lactobacillus*, *Corynebacterium*, and *Staphylococcus* as active species in mice, with *Escherichia* being the only genus shared with humans. Experiments with germ-free mice colonized with human gut microbiota confirmed similar BBN metabolism and BCPN production. Additionally, gut microbiota influenced the toxicokinetic of other nitrosamine carcinogens, highlighting a potential strategy to target microbiota for reducing cancer risk associated with chemical exposures. In the future, modulation of the microbiome could represent a viable approach to reduce BC predisposition and serve as a preventative intervention against carcinogenesis [223]. However, no studies have yet demonstrated the role of gut microbiota in sex-biased BC.

### Metabolism

Male and female metabolisms differ significantly, but their role in cancer, especially in the metabolic reprogramming of cancer cells, has only recently been explored. Carcinogen metabolism is closely related with BC, including nitrosamine ketones derived from nicotine, polyaromatic hydrocarbons (PAHs) from cigarette smoke, and arsenic [224]. To detoxify these carcinogens, multiple CYP450 enzymes are involved and encoded by *CYP1B1*, *GSTM1*, and *GSTP1* [225, 226]. Genetic polymorphisms in *CYP1B1*, *GSTM1*, and *GSTP1* are associated with bladder cancer risk [227]. Sex hormones have been shown to regulate expression of these enzymes [228]. Researchers found that the reduction of the tobacco carcinogen 4-methylnitrosamino-1–3-pyridyl-1-butanone (NNK) to 4-methylnitrosamino-1–3-pyridyl-1-butanol (NNAL)—a key detoxification step mediated by SDR enzymes (CBR1, 11βHSD1) and AKR enzymes (AKR1B10, AKR1 C1, AKR1 C2, AKR1 C4)—is more strongly inhibited by female sex hormones and contraceptives (estradiol, progesterone, ethinylestradiol, drospirenone) than by testosterone, highlighting sex-specific differences in carcinogen metabolism [229, 230]. Female BC patients express more progesterone than male [231]. In female BC patients, progesterone may impair the removal of toxins from the body by inhibiting the CYP450 enzymes. Sex differences have been observed in the biotransformation of various molecules, including hormones, neurotransmitters, drugs, and xenobiotics, impacting their response to environmental exposures, usage, accumulation, and elimination. A recent study by, Zheng et al., revealed that a single mutation at the His213 allele of a sulfotransferase gene (*SULT1 A1*) conferred decreased BC risk only in females [232]. The UDP-glucuronosyltransferases (UGT)-dependent detoxification pathway eliminates xenobiotics and endobiotic [233]. Human UGT loci have been closely linked to BC [234]. The expression of the UGT enzyme, UGT2B17 in men is higher [235]. Interestingly, ARsignaling represses the expression of UGTs in the bladder and prostate, suggesting a strong sex biased role for the UGT detoxification pathway in the bladder [236] and prostate [237]. It has been shown that downregulation of UGTs is closely associated to tumor formation in mice [238] and humans [237, 239]. Thus, males and females have different capacities to metabolize carcinogens and chemotherapeutics.

Mitochondria, another critical player in metabolism, are maternally inherited and exhibit strong sexual-specific activity in normal and pathological conditions [240]. One of the outputs of mitochondrial activity is reactive oxygen species (ROS), and it has been suggested that female mitochondria have a better ability to maintain lower ROS levels than male mitochondria in the brain [241]. It remains to be shown, however, whether this phenomenon also affects male and female bladders.

## Omics, AI, and sex-specific biomarkers: decoding sex differences

Discovering reliable biomarkers and delineating the complex mechanisms from omics data contribute to precision medicine among individuals and between sexes [117, 155, 156]. For example, RNA sequencing and whole exon sequencing can illuminate the relationship between sex-specific gene expression and genetic anomalies in a patient cohort [117, 242]. The recent development of single-cell and spatial technologies enhances our ability to explore the biomarkers and underlying mechanisms of BC at the single-cell level [243–245]. For example, single-cell RNA sequencing (scRNA-seq) enables the identification of distinct cellular subpopulations that play roles in tumor aggression or treatment resistance [246]. Additionally, this technology is instrumental in unraveling how abnormalities in sex chromosomes affect cellular functionality and fate. This includes studying the impacts of the functional silencing of sex chromosomes, allowing researchers to link these genomic changes with specific alterations in cell transcriptional behavior across different cell types [135].

At the same time, spatial omics help researchers map these findings within tumor spatial components, offering clues about the TME that could influence disease progression and response to treatment [247, 248]. By enabling the dissection of cellular heterogeneity and the spatial organization of cells within the bladder tumor microenvironment, these technologies have uncovered insights into the sex-biased molecular drivers in altering cell neighborhood, differentiation, and functions. We anticipate that implementing large-scale single-cell and spatial omics data from BC could improve our understanding of sex differences in BC [249–251].

A study using scRNA-seq and spatial transcriptomics revealed sex-specific differences in the tumor microenvironment. Females showed upregulation of genes related to bacterial response and apoptosis, while males had higher ribosome biogenesis and MYC activity, with more urothelial cells in active cell cycle phases. This suggests enhanced barrier defense but potentially increased tumor risk in males due to p53 degradation. Female fibroblasts had higher collagen gene expression [252]. In mouse and human studies of BC using scRNA-seq, sex-specific differences in the TME were observed. Males showed higher activity in glycolysis, gluconeogenesis, PKA, and PI3 K/AKT signaling, while females had increased thrombin, endocannabinoid, and oxytocin signaling, along with higher expression of tumor suppressors EGR2 and EGR3 [47]. Female patients also had more CD163⁺ macrophages and B cells, with M2-like macrophages producing CXCL13 upon IL-10 and LPS stimulation. These immune cell populations were linked to poorer recurrence-free survival in both sexes [253, 254].

Study on patients presenting with hematuria demonstrated that blood and urine biomarkers for assessing BC risk exhibit sex-specific differences and developed distinct biomarker algorithms for males (u_NSE, s_PAI-1/tPA, u_midkine, u_NGAL, u_MMP-9/TIMP-1, and s_prolactin) and females (IL-12p70, IL-13, midkine, and clusterin), which showed high sensitivity and specificity, with areas under the receiver operating characteristic curve (AUROC) of 0.795 for males and 0.865 for females [255]. The incorporation of clinical variables, such as infection, further improved the AUROC to 0.822 for males and 0.923 for females, highlighting the potential for integrating biomarkers with clinical data to enhance diagnostic accuracy [255]. However, a key limitation is the lack of large-scale, prospective clinical trials that validate these findings across diverse populations and assess their long-term impact on patient outcomes, particularly in terms of how these sex-specific approaches translate into improved survival rates and quality of life for both male and female patients. In addition, we also face a range of computational challenges. These include integrating different modalities that often display varying levels of sparsity, handling the high dimensionality of datasets, and mitigating the impact of noise across multiple samples. We emphasize the potential of graph-based machine learning to effectively navigate these challenges by leveraging the complex relationships within the data, thus enabling a more robust and integrated analysis [256–259]. Moreover, we envision the development of sophisticated frameworks capable of integrating and synthesizing information from fundamental biological research to clinical diagnosis and prognosis. AI-driven foundation models, trained on manually curated multi-modal data, emerge as a promising tool [260, 261]. These models provide an end-to-end solution that interprets and integrates large-scale, diverse omics datasets, such as sequencing and imaging technologies [262]. They are crafted not only to deepen our understanding of BC biology [263–265] but also to enhance diagnostic accuracy and prognostic precision [266–268]. By utilizing these advanced technologies, healthcare providers can develop treatment strategies that are finely tailored and responsive to the unique biological characteristics of each patient. This enhanced personalization significantly improves the ability to predict and monitor disease progression and treatment response, leading to improved clinical outcomes for BC.

## Future directions

In this review, we emphasized the distinct biological traits between males and females, but it’s crucial to recognize that even within the same biological sex, individual variations do exist. For instance, certain sex-biased gene regulators may vary significantly between individuals of the same sex, complicating simple male–female comparisons. Moreover, an analysis of the National Cancer Database revealed that transgender patients with BC had significantly worse overall survival compared to their cisgender counterparts, with a hazard ratio of 2.86 (95% CI: 1.36–6.00), indicating nearly a threefold higher risk of death [269]. Consequently, future research should go beyond binary sex comparisons, such as examining the spectrum of sex-specific effects, isolating individual sex-biasing factors to avoid confounding, assessing potential interactions between factors, and accounting for sex-specific, age-related changes such as hormone declines. We believe that integrating –omics molecular and patient data and leveraging emerging AI technologies will enable researchers to dissect these complex biological interactions, ultimately improving BC outcomes for all patients through more precise and personalized care.

## Data Availability

No datasets were generated or analysed during the current study.

## Abbreviations

BC: Bladder cancer
NMIBC: Non-muscle-invasive bladder cancer
MIBC: Muscle-invasive bladder cancer
FGFR3: Fibroblast growth factor receptor 3 (FGFR3)
CDKN2 A: Cyclin-dependent kinase inhibitor 2A
TP53: Tumor protein p53
RB1: Retinoblastoma
TERT: Telomerase reverse transcriptase
PD-L1: Programmed cell death ligand-1
SABV: Sex as a biological variable
TURBT: Transurethral resection of bladder tumor
CIS: Carcinoma in situ
BCG: Bacille-Calmette Guerin
UTI: Urinary tract infection
UBC: Urinary bladder cancer
FXR: Farnesoid X receptor
SHP: Small heterodimer partner
TME: Tumor microenvironment
GPER: G protein-coupled estrogen receptor
EGFR: Epidermal growth factor receptor
MAPK: Mitogen-activated protein kinase
PKA: Protein kinase A
PI3 K: Phosphoinositide 3-kinase
PR: Progesterone receptors
AR: Androgen receptor
NR3 C4: Nuclear receptor subfamily 3, group C, member 4
BBN: *N*-Butyl-*N*-(4-hydroxybutyl) nitrosamine
MDSCs: Myeloid-derived suppressor cells
SNVs: Single nucleotide variants
